# Butterfly species diversity and their floral preferences in the Rupa Wetland of Nepal

**DOI:** 10.1002/ece3.7177

**Published:** 2021-02-03

**Authors:** Bandana Subedi, Alyssa B. Stewart, Bijaya Neupane, Sudha Ghimire, Hari Adhikari

**Affiliations:** ^1^ Institute of Forestry Tribhuvan University Pokhara Nepal; ^2^ Department of Plant Science Mahidol University Bangkok Thailand; ^3^ Earth Change Observation Laboratory Department of Geosciences and Geography University of Helsinki Helsinki Finland; ^4^ Faculty of Science Institute for Atmospheric and Earth System Research University of Helsinki Helsinki Finland

**Keywords:** corolla depth, Lepidoptera, nectar plants, proboscis length, species diversity

## Abstract

Floral attributes often influence the foraging choices of nectar‐feeding butterflies, given the close association between plants and these butterfly pollinators. The diversity of butterflies is known to a large extent in Nepal, but little information is available on the feeding habits of butterflies. This study was conducted along the periphery of Rupa Wetland from January to December 2019 to assess butterfly species diversity and to identify the factors influencing their foraging choices. In total, we recorded 1535 individuals of 138 species representing all six families. For our examination of butterfly–nectar plant interactions, we recorded a total of 298 individuals belonging to 31 species of butterfly visiting a total of 28 nectar plant species. Overall, total butterfly visitation was found to be significantly influenced by plant category (herbaceous preferred over woody), floral color (yellow white and purple preferred over pink), and corolla type (tubular preferred over nontubular). Moreover, there was a significant positive correlation between the proboscis length of butterflies and the corolla tube length of flowers. Examining each butterfly family separately revealed that, for four of the families (Lycaenidae, Nymphalidae, Papilionidae, and Pieridae), none of the tested factors (flower color, plant category, and corolla type) were shown to significantly influence butterfly abundance at flowers. However, Hesperidae abundance was found to be significantly influenced by both flower color (with more butterflies observed at yellow flowers than purple) and flower type (with more butterflies observed at tubular flowers than nontubular flowers). Our results reveal that Rupa Lake is a suitable habitat for butterflies, providing valuable floral resources. Hence, further detailed studies encompassing all seasons, a greater variety of plants, and other influential factors in different ecological regions are fundamental for creating favorable environments to sustain important butterfly pollinators and help create balanced wetland ecosystems.

## Graphical abstract

We assessed the number of butterfly species, their abundance, and their floral foraging behavior, from 28 linear transects (500 m long each) placed in a stratified and random manner throughout the study area. Five factors, that is, category of plant, flower color, corolla shape, corolla depth, and the proboscis length of butterfly species, were taken into account to assess the nectar plant choices of butterfly families. Moreover, species diversity at the family level, and overall, was determined through several indices.

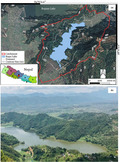

## 1 INTRODUCTION

Nepal is remarkably diverse in flora and fauna due to its climatic and topographical variation. The dramatic differences in elevation and microclimate result in a variety of ecosystems, from tropical savannas along the Indian border to subtropical broad leaf and coniferous forests in the Hilly region and to temperate broadleaf and coniferous forests on the lap of the Himalayas (MoFE, 2018; Tripathi et al., 2020). Indeed, previous work in mountainous areas has shown that topological complexity promotes high species richness (Zhang et al., 2020). Nepal provides another example, as its land area occupies just 0.1% of global area, but it contains 3.2% of the world’s floral diversity and 1.1% of global faunal diversity (MFSC, 2014).

Insects are one of the key indicators of healthy ecosystems, and they play a significant role in ecosystem functioning (Springett, 1978). Butterflies, one of the best‐known pollinators and bio indicators, belong to the order Lepidoptera (suborder Rhopalocera) (Durairaj & Sinha, 2015). Out of around 20,000 species of butterflies recorded worldwide, Nepal is home to 672 species from 263 genera, which is about 4.3% of globally known species (Akram et al., 2018; Panthee et al., 2019; Poel, 2020; Pohl et al., 2011; Sajan, 2020; Sajan & Pariyar, 2019; Sapkota et al., 2020; Smith, 1981; Smith, 1989; Smith & Majupuria, 2006; Tamang, Nuppa, et al., 2019). Around 29 species and subspecies of butterflies are endemic to Nepal. These endemic species are disappearing slowly, and about 18% of the butterfly species found in the midhill zones are considered threatened (ICIMOD, 2007). A total of 142 species of butterflies found in Nepal are under the IUCN red list category, among which 12 are endangered, 43 are vulnerable, and 87 are susceptible (Paudel et al., 2012). Likewise, three species (*Teinopalpus imperialis*, *Troides aeacus*, and *Troides helena*) are placed under CITES Appendix S2 (Khanal et al., 2013).

The evolution of angiosperm is closely associated with insect pollinators that influence their reproductive success (Sargent & Ackerly, 2008; Wright & Schiestl, 2009). Butterflies are phytophagous insects that feed on nectar and occasionally pollen. Their sectorial proboscis performs the major function of feeding, and in doing so they often contribute to pollination (Bauder et al., 2013; Bauder et al., 2011; Blüthgen & Klein, 2011). While there is differential exploitation of flowering plants among butterfly species, they tend to be opportunistic generalists (Courtney, 1986). Usually, the floral preferences of butterflies are influenced by flower color, nectar concentration, nectar quantity and quality, flower structure, flower shape, and size (Boggs & Ross, 1993; Erhardt, 1991; Ilse, 1928; Pohl et al., 2011; Tiedge & Lohaus, 2017). Additionally, foraging preferences also depend upon the compatibility between flower morphology (i.e., corolla length) and butterfly morphology (i.e., proboscis length) (Bergerot et al., 2010; Tiple et al., 2009).

While insects are known to be critical to ecosystem functioning, the biodiversity of insects is threatened worldwide. There has been a dramatic decline among Lepidopterans that may lead to the extinction of 40% of species over the next few decades (Sánchez‐Bayo & Wyckhuys, 2019). Minor changes in their habitat may lead to either migration or local extinction if the required attention is not given (Kunte, 1997) because many species require specific plants as food or sites for reproduction (Bernays & Graham, 1988). The biggest threat that humans pose to the survival of insects, including butterfly populations, is habitat destruction (New et al., 1995). Due to the rapid increase in global human population size, anthropogenic changes are impacting butterflies through both direct habitat loss as well as the loss of plant species on which butterflies depend (Hoyle & James, 2005). Moreover, butterflies are particularly sensitive to environmental changes (Stefanescu et al., 2011), including the fast rise of industries, intense use of fertilizers and insecticides, climate change, nitrogen pollution, mono‐cropping, forest fires, fragmentation, and habitat degradation, all of which make them vulnerable to extinction. As butterflies are known to be flagship species for insect conservation (Tiple et al., 2005; Wagner et al., 2003), any research efforts that target the conservation of butterfly species will automatically save many other species in the area. To protect this flagship group from further population declines, and potential species extinctions, studies examining their diversity, habitat suitability, and nectar plant choices are necessary.

Studies on butterflies in the Eastern Himalayas are focused mostly on their diversity in protected areas or the forest ecosystem (Chettri, 2015; Chettri et al., 2018; Dewan et al., 2019; Kumar Acharya & Vijayan, 2011; Sharma et al., 2020), whereas few ecological studies of butterflies are conducted in relation to their nectar or larval host plants (Ghosh & Saha, 2016; Sengupta & Ghorai, 2013). Many studies of butterflies in the Western and Southern Himalayas have recorded a high diversity of butterflies (Arya & Dayakrishna, 2014; Kumar et al., 2017; Singh & Sondhi, 2016; Tyagi et al., 2011; Uniyal & Mathur, 1998) and a few more studies have examined butterfly‐plant interactions (Arya et al., 2020; Nimbalkar et al., 2011; Tiple et al., 2005, 2009). While there have been many studies on butterflies from different parts of Nepal (in the Central Himalayan region) (Bhusal & Khanal, 2008; Khanal, 2006; Khanal et al., 2013, 2014; Rai, 2017; Smith & Majupuria, 2006; Suwal et al., 2019), previous studies have focused on the diversity, taxonomy, and distribution of butterflies, and few studies have examined butterfly‐plant interactions (Nepali et al., 2018; Shrestha et al., 2020). However, extensive ecological studies to determine the factors that influence butterfly foraging choices are crucial to improve the ecological utility of butterflies and to preserve them as indicator taxa (Zhang et al., 2020).

Given the lack of sufficient knowledge about butterfly diversity and their floral foraging preferences in Nepal, this research aimed to fulfill this gap by addressing two main objectives. The first objective was to examine butterfly diversity and abundance throughout the year at Rupa Wetland. This area is known to support high butterfly diversity (Smith et al., 2016), but we still lack long‐term studies (spanning multiple seasons) that quantify the abundance of different species. The second objective was to examine butterfly–nectar plant interactions and to assess the factors influencing floral foraging choices. The information gained from our two objectives is necessary to conserve both butterflies and their preferred nectar plants in an effective and sustained manner. The present study emphasizes the importance of nectar sources to insects. While the present work is only a preliminary study, it provides a solid foundation for future studies, which are needed to understand in greater detail the utility of butterflies to flowers and vice versa. Indeed, our findings raise a host of focal questions that should be addressed in the coming years.

## 2 MATERIALS AND METHODS

### 2.1 Study area

**FIGURE 1:**
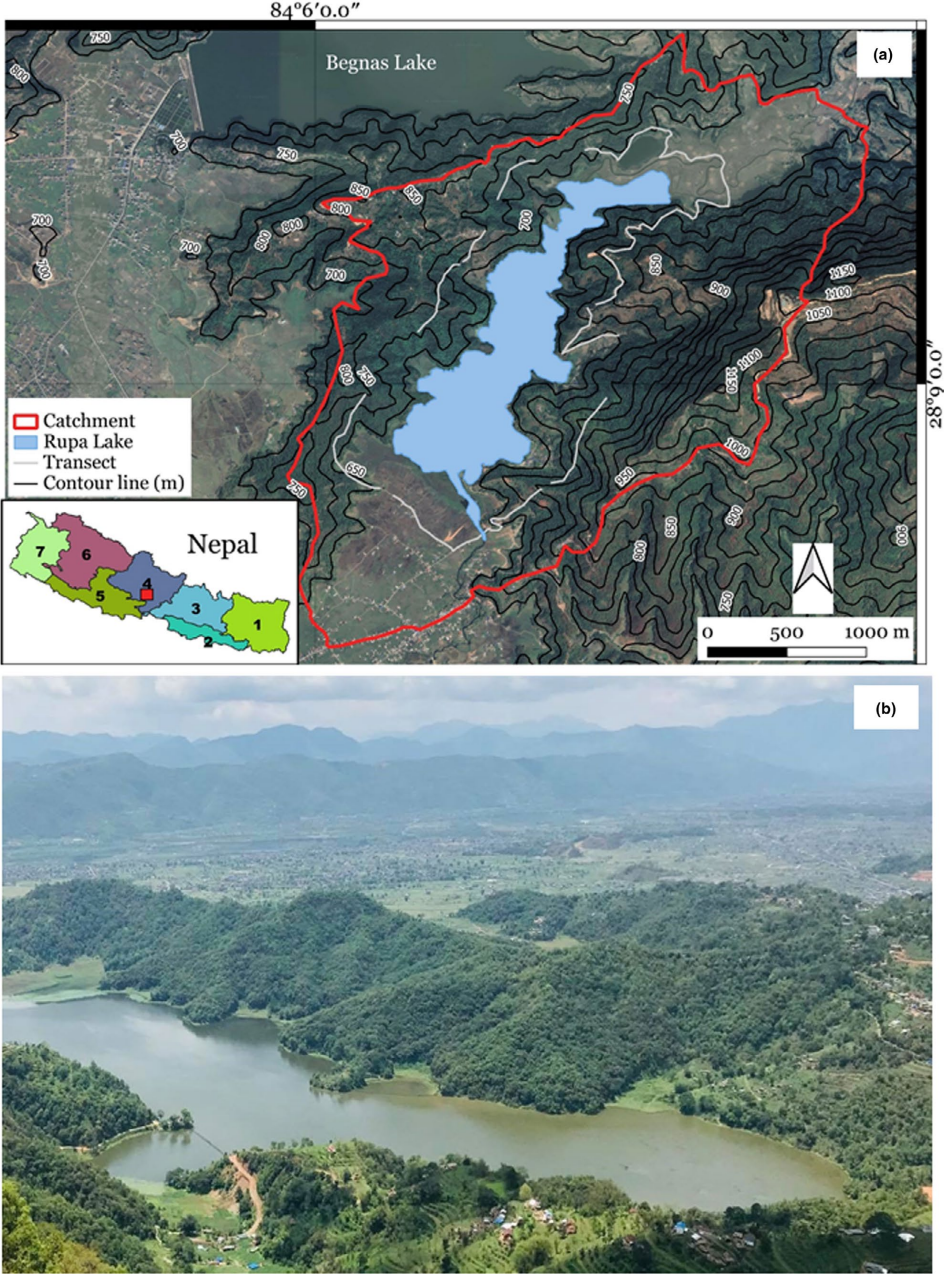
(a) Geographic location of the study area shown on Rapid eye image from March 16, 2019; Rupa Lake is outlined in blue, the catchment area is outlined in red, and white lines indicate the locations of study transects. (b) A photograph of Rupa Wetland, showing the land use and land cover types in the study area (@Damodar Bhakta Thapa)

Rupa Wetland (28°8′55″N 84°6′40″E) (Figure 1), declared a Ramsar site in February 2016, is one of the most important wetlands of Nepal situated in Chitwan Annapurna Landscape at an elevation of 600 m above sea level (Paudel et al., 2017). The lake serves as a famous tourist destination and also supports fish farming, thus providing a great source of income to local livelihoods (Rajbhandari & Shrestha, 2014). It is the third biggest lake of Pokhara valley with a total watershed area of 3,000 hectares and a lake area of 112 ha. The total catchment area of Rupa Wetland is around 30 km^2^. The lake provides suitable habitat for diverse butterflies, dragonflies, fish species, and some major wetland bird species (Gautam et al., 2019). The wetland is important for migratory birds, and 36 species of water birds have been recorded (Kafle et al., 2008). This study was conducted in the catchment area of the lake. It constitutes 361 species of vegetation and 175 species of forest medicinal herbs (Dangol, 2015). Many of the plant species found in the study area are ornamentals (of both native and exotic species) that are often intentionally cultivated for their showy flowers (B. Subedi, pers. obs.).

The eastern portion of Rupa Lake is covered with mixed forests, predominantly consisting of Chilaune (*Schima wallichii*) and Katus (*Castanopsis indica*), and western portion is partially covered with vegetation and cultivated land. The northern slopes consist of privately owned terraced lands that are cultivated with agricultural crops, and some floating aquatic vegetation, grasses, and rice fields are found along the lake shoreline. The area holds global significance, as it is an internationally important Ramsar site that harbors high biological diversity, particularly numerous wetland species including migratory birds and butterfly species. Additionally, Rupa Lake is the only lake where payment for ecosystem services (PES) has been implemented and upstream forest user groups are compensated for their conservation efforts. A certain percentage of the net income from the lake are paid to the upland communities through a PES system (Regmi et al., 2009).

### 2.2 Data collection

The study area was fully explored from January to December 2019 throughout Nepal’s four seasons: premonsoon (March to May), monsoon (June to September), postmonsoon (October to November), and winter (December to February; Devkota, 2014). To address our first objective (assessing butterfly diversity and abundance), we collected data from March to November 2019; data was not collected during the winter due to the lack of butterflies during this season, as they are intolerant to cold temperatures (McDermott Long et al., 2017). To address our second objective (examining butterfly foraging choices), we collected data from February to July 2019, which covered the flowering periods of diverse plant species in the study area. We started observing plant–butterfly interactions in February, when the first flowers started to appear, but since butterflies were still scarce in February, we did not begin assessing butterfly diversity and abundance until March. While butterfly diversity and abundance were assessed through November, limited manpower required to us to end plant–butterfly observations in July; further research should carry out observations through the end of the flowering season.

Data for both objectives were collected using the transect count method described by Pollard (1977). A total of 28 transects, 500 m long each, were arranged in a stratified and random manner at an interval of 100 m apart, at a distance of 1 km from the lake’s edge (i.e., scrubland where maximum butterflies were observed) (Figure 1a). Each transect was walked at a slow, constant pace and all butterflies within 5 m of the observer walking the transect (to either side, in front, and above) were counted and recorded. It is possible that some butterflies in the transects were counted more than once. However, based on our observation, most individuals forage at the same patch for a long period and therefore we are confident that most of the butterflies recorded were unique observations. Each transect was walked twice per month, resulting in a total of 18 replicates for the butterfly diversity and abundance data, and 12 replicates for the butterfly foraging data. Transect lines were walked in the morning between 8:00 to 12:00 h on sunny days (avoiding rainy and windy days) so that maximum butterfly species could be spotted (Caldas & Robbins, 2003). Butterflies were identified in the field based on their behavioral and morphological characteristics following Smith and Majupuria (2006) and plants were identified based on leaf, floral, and fruit characteristics following Storrs and Storrs (1990).

Additionally, for data collected on butterfly foraging choices, attempts were made to catch every feeding butterfly seen on each transect by using a sweep net. Proboscis length was determined by restraining the tip of the unfurled proboscis with forceps or a needle and measuring the distance from the base to the tip (Ehrlich & Raven, 1964; Kunte, 2007; Sultana et al., 2017). Moreover, the flower corolla at which the butterfly was observed was plucked to measure the corolla tube length. Corolla depth was measured from the most convenient point from which a butterfly might place the proboscis to the corolla base, where the nectar was available. For each plant species, we also recorded plant category (herbaceous or woody), flower color, and corolla type (tubular or nontubular). Finally, for the butterfly diversity and abundance data, we used the number of butterfly sightings to categorize each species as very rare (<2 sightings), rare (2–15 sightings) not rare (15–50 sightings), common (50–100 sightings) and very common (>100 sightings) to determine the site‐specific status of each butterfly species (Shrestha et al., 2018; Tiple et al., 2005).

### 2.3 Data analysis

We calculated the Shannon–Weiner diversity index (Shannon & Weaver, 1949), Simpson Index (Simpson, 1949), species richness, Pielou’s evenness (Pielou, 1966), Margalef’s richness index (Margalef, 1958), and relative abundance of each butterfly family to quantify butterfly diversity in the Rupa wetland. The Shannon–Weiner diversity index provides information about the community composition of species; the higher the number, the higher the species diversity. Simpson’s index is a weighted arithmetic mean of proportional abundance and measures the probability that two individuals randomly selected from a sample will belong to the same species. It is a dominance index because it gives more weight to common or dominant species, whereas the Shannon–Weiner index gives more weight to rare species. Simpson’s index ranges from to 0 to 1 with 0 representing infinite diversity and 1 representing no diversity, so the larger the value of D, the lower the diversity. Species richness denotes the total number of species observed within an area. Margalef’s index was used as a simple measure of species richness (Margalef, 1958) and Pielou’s evenness index (e) was used for calculating the evenness of species (Pielou, 1966). Species abundance denotes the total number of individuals observed during the study period.

For our butterfly‐plant interaction data, we used generalized linear modeling (GLM) (Nelder & Wedderburn, 1972) to identify the factors affecting nectar plant choice by butterflies. Butterfly species abundance was used as the dependent variable whereas flower color, plant category, and corolla type were used as independent variables with a Poisson distribution. We used nested likelihood ratio tests (Neyman & Pearson, 1933) to choose the best model, followed by Turkey’s post hoc tests (Tukey, 1949) in the case of significant predictors. Differences were considered significant at *p* < 0.05. Additionally, Pearson’s correlation coefficient was used to test for a significant relationship between the proboscis length of butterflies and the corolla depth of flowers. All butterfly‐plant analyses were conducted in R 4.0.2 (R Core Team, 2019).

## 3 RESULTS

### 3.1 Species diversity and abundance trends of butterflies

Altogether, 1,535 individuals of 138 species representing all six families of butterflies were counted and recorded in the single wetland. Among them, Punchinello (*Zemeros flegyas,* 92 individuals) and Grey pansy (*Junonia atlites,* 80 individuals) butterflies were the most abundant species, followed by Straight swift (*Parnara guttata,* 69 individuals), Common five ring (*Ypthima baldus,* 45 individuals), and Common grass yellow (*Eurema hecabe,* 38 individuals) butterflies. The least common species included Pioneer (*Belonois aurota,* 1 individual), Common birdwing (*Troides helena,* 1 individual), Tree yellow (*Gandaca harina,* 1 individual), Pale Wanderer (*Pareronia avatar,* 2 individuals), Yellow orange tip (*Ixias pyrene,* 2 individuals), Peablue (*Lampides boeticus,* 3 individuals), Chocolate albatross (*Appias lyncida,* 4 individuals), Dark cerulean (*Jamides bochus,* 5 individuals), and Dark pierrot (*Tarucus ananda,* 8 individuals) butterflies (Annex 1).

**FIGURE 2:**
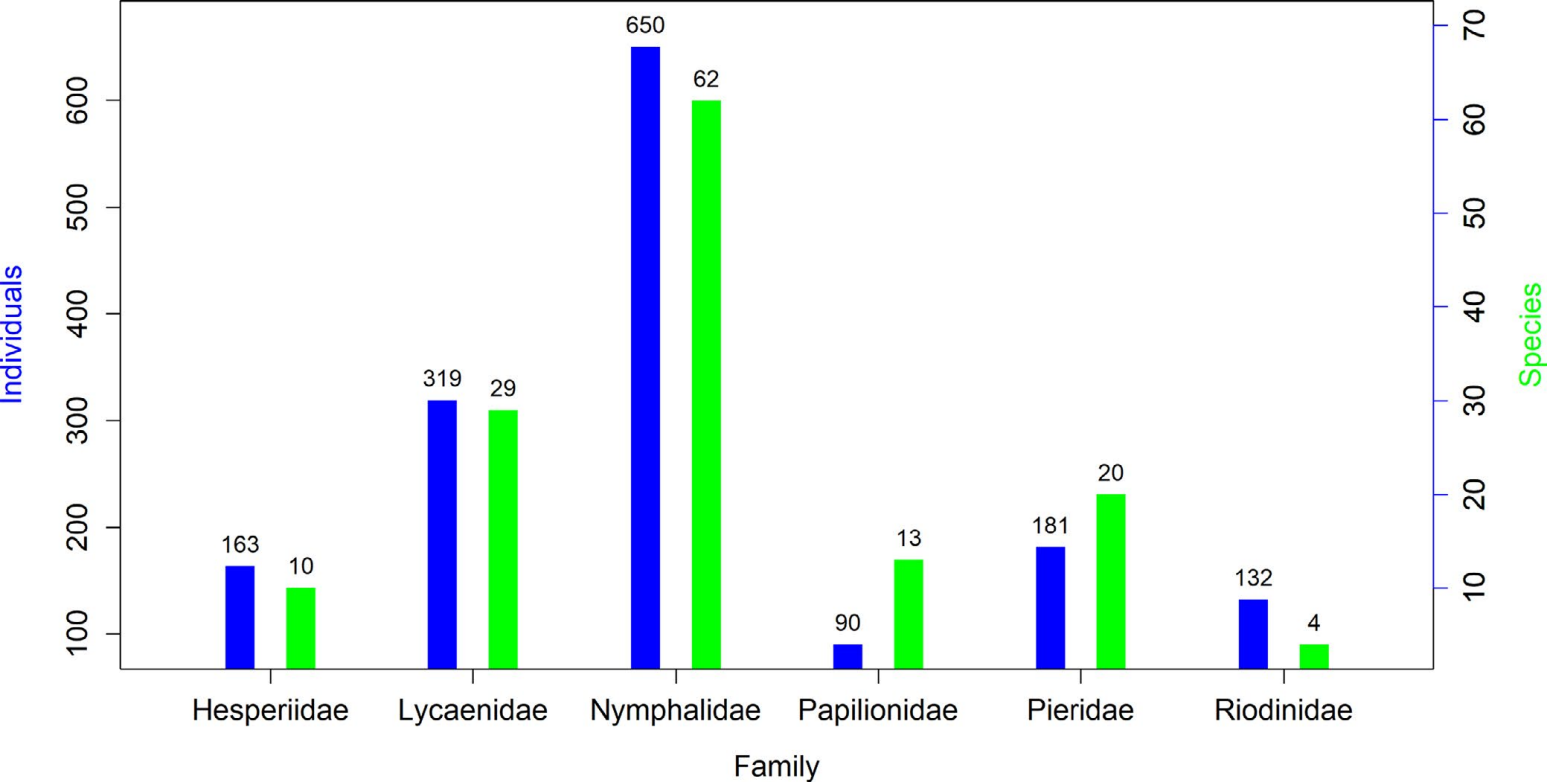
The number of individuals and species of each butterfly family observed in Rupa Wetland, Nepal

The family with the most observed individuals was the Nymphalidae family (650 individuals of 62 spp), followed by Lycaenidae (319 individuals of 29 species), Pieridae (181 individuals of 20 species), Hespiridae (163 individuals of 10 species), Riodinidae (132 individuals of 4 species), and Papilionidae (90 individuals of 13 species) (Figure 2).

In the study area, we observed 3 common, 29 not rare, 61 rare, and 45 very rare butterfly species (Annex 1). Family Nymphalidae had the highest Shannon diversity index of 3.50 while family Riodinidae showed the lowest diversity with a value of 0.84. The overall Shannon diversity index, Simpson’s index, species richness, Pielou evenness, and Margalef richness index of butterfly fauna in Rupa wetland (pooling all families) were 4.33, 0.98, 138, 0.87, and 18.67, respectively. Diversity indices for each family in the Rupa Wetland are summarized in Table 1.

**TABLE 1:** Descriptive measures of diversity (Shannon–Weiner diversity index, Simpson diversity index, species richness, Pielou’s evenness, and Margalef’s richness index) calculated for each butterfly family observed in Rupa Wetland, Nepal, as well as the overall values when data from all families were pooled together

Family	Shannon Index	Simpson Index	Species richness	Pielou evenness	Margalef’s Richness Index
Hesperiidae	1.72	0.76	10.00	0.75	1.23
Lycaenidae	2.99	0.94	29.00	0.89	3.82
Nymphalidae	3.50	0.96	62.00	0.85	8.31
Papilionidae	2.11	0.85	13.00	0.82	1.64
Pieridae	2.48	0.89	20.00	0.83	2.59
Riodinidae	0.84	0.46	4.00	0.60	0.41
All families	4.33	0.98	138	0.87	18.67

### 3.2 Effects of floral traits on butterfly abundance

**FIGURE 3:**
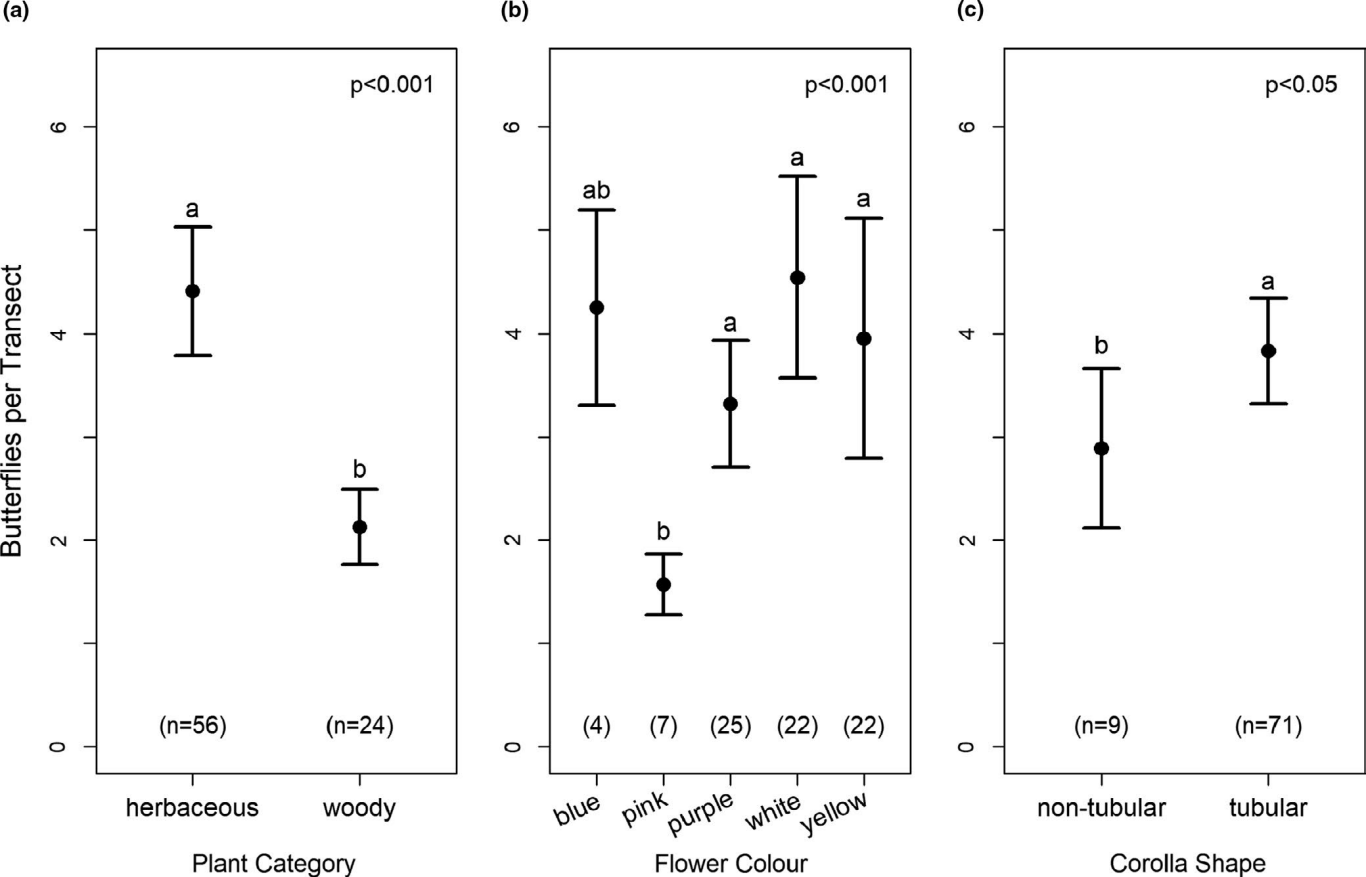
The mean (±*SE*) number of butterflies observed per transect at (A) the flowers of herbaceous versus woody plant species, (B) different floral colors, and (C) nontubular versus tubular flowers. Within each graph, traits with different lowercase letters are significantly different (Tukey’s post hoc test, *p* < 0.05). Numbers in parentheses at the bottom of each graph indicate the sample sizes (number of sightings)

**FIGURE 4:**
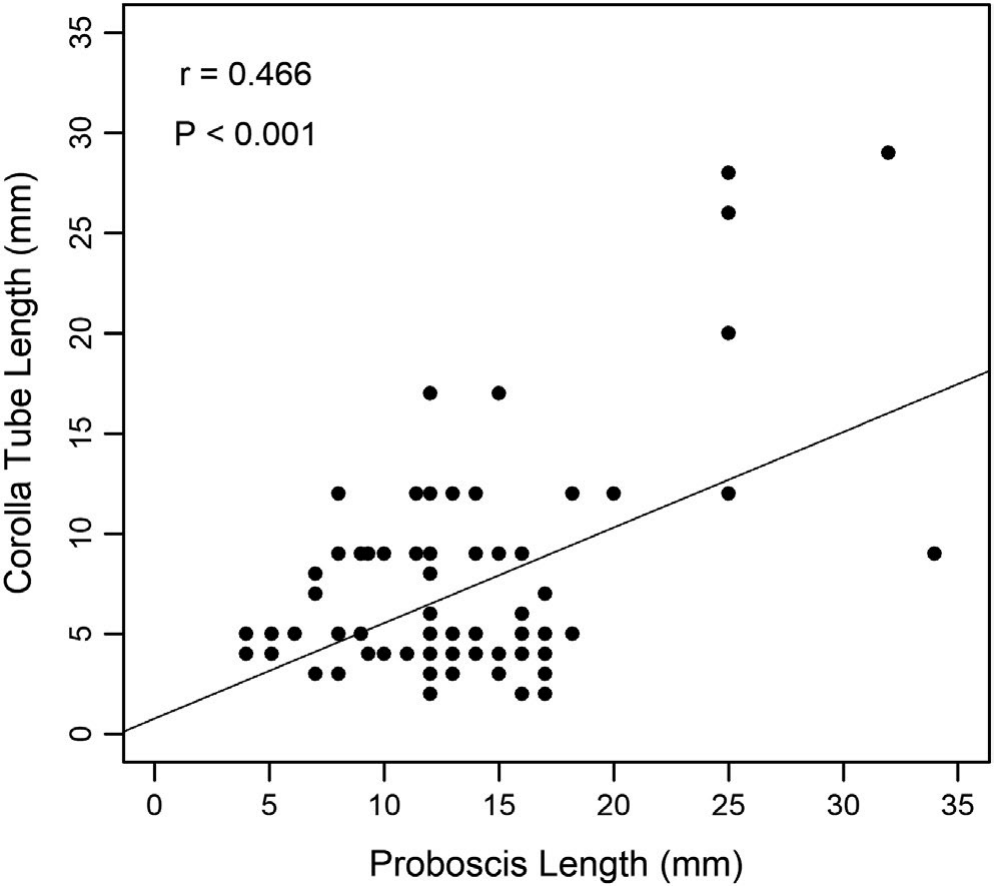
A scatterplot showing the significant positive correlation between butterfly proboscis length and corolla tube length of the flowers they foraged at in Rupa Wetland, Nepal

Out of the 138 butterfly species observed, only 31 species consisting of 298 individuals were observed feeding at flowers; they were recorded at a total of 28 nectar plant species. When all 31 butterfly species were analyzed together, results of the GLM revealed that butterfly visitation was significantly influenced by plant category ($\chi_{1}^{2}$ = 0.50, *p* = 0.48), flower color ($\chi_{4}^{2}$ = 12.3, *p* = 0.015), and corolla type ($\chi_{1}^{2}$ = 1.22, *p* = 0.27) (Figure 3). Butterflies significantly preferred the flowers of herbaceous plants over woody plants (Figure 3A), and tubular flowers over nontubular flowers (Figure 3C). Moreover, Tukey’s test revealed that butterfly abundance was significantly greater at yellow, white, and purple flowers than at pink flowers (*p* < 0.05; Figure 3B). Moreover, our results show a significant correlation between the proboscis length of butterflies and the corolla tube length of visited flowers (*p* < 0.001, *r* = 0.466; Figure 4). The shortest mean proboscis length was 7.10 mm for Lycaenidae and the longest was 25.71 mm for Papilionidae (Appendix S3). Similarly, the shortest mean corolla tube length was 4.38 mm for flowers visited by Hesperidae butterflies and the longest was 19.43 mm for flowers visited by Papilionidae butterflies.

**FIGURE 5:**
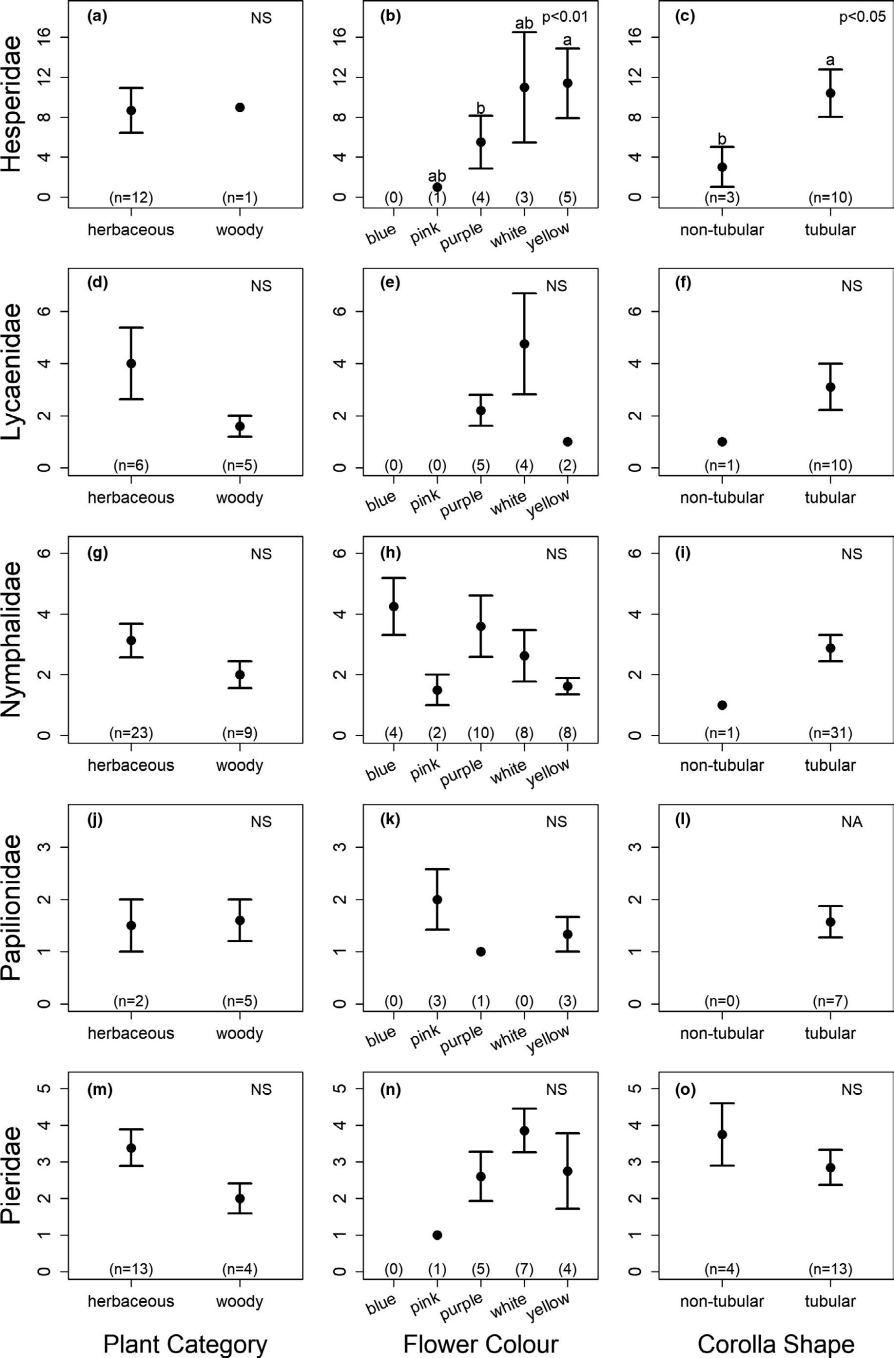
The mean (±*SE*) number of butterflies observed per transect for each of 5 butterfly families observed: (a–c) Hesperidae, (d–f) Lycaenidae, (g–i) Nymphalidae, (j–l) Papilionidae, and (m–o) Pieridae. Graphs show the number of butterflies at (a, d, g, j, m) the flowers of herbaceous versus woody plant species, (b, e, h, k, n) different floral colors, and (c, f, i, l, o) nontubular versus tubular flowers. Within each graph, different lowercase letters indicate significant differences. Numbers in parentheses at the bottom of each graph indicate the sample sizes (number of sightings)

Examining each butterfly family separately revealed different results (Figure 5). For four of the families (Lycaenidae, Nymphalidae, Papilionidae, and Pieridae), none of the tested factors (flower color, plant category, and corolla type) were shown to significantly influence butterfly abundance at flowers (Figure 5D–O). However, Hesperidae abundance was found to be significantly influenced by both flower color ($\chi_{3}^{2}$ = 12.1, *p* = 0.007), with more butterflies observed at yellow flowers than purple flowers (Figure 5B), and flower type ($\chi_{1}^{2}$ = 5.78, *p* = 0.02), with more butterflies observed at tubular flowers than nontubular flowers (Figure 5C).

### 3.3 Number of butterfly species feeding at nectar plant species

**FIGURE 6:**
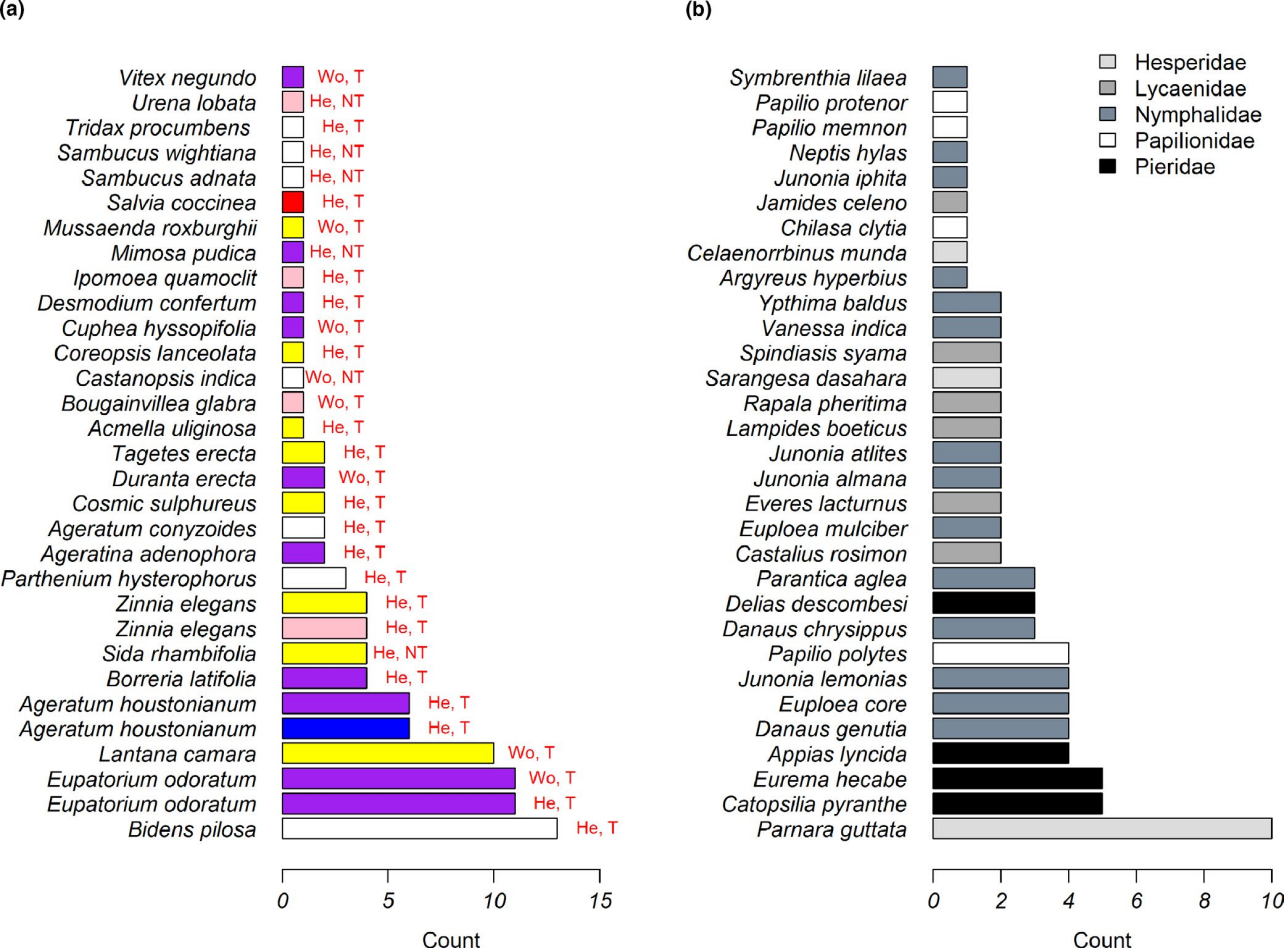
(a) The number of butterfly species visiting each observed plant species. The color in the bar plot represents flower color; Wo, He, T, and NT represent woody, herbaceous, tubular, and nontubular, respectively. (b) Number of plant species visited by each butterfly species

To ascertain the popularity of nectar plant species, the total number of butterfly species observed feeding on each plant species was counted. Twenty‐eight nectar host plant species were observed receiving butterfly visits. *Bidens pilosa* was visited by the most butterfly species (13 species), followed by *Eupatorium odoratum* (11 species), *Lantana camara* (10 species), and *Ageratum houstonianum* (6 species); 15 plant species were visited by a single butterfly species (Figure 6A). *Parnara guttata* butterflies visited the most plant species (10 species), followed by *Catopsilia pyranthe* (5), Eurema hecabe (5), and *Appias lyncida* (4), whereas 9 butterfly species visited only a single plant species (Figure 6B).

## 4 DISCUSSION

A total of 1,535 individuals from 138 species representing all six families of butterflies were counted and recorded in the single wetland. Similarly, Smith et al. (2016) recorded a total of 174 butterfly species in Rupa and Begnas Lakes. As mentioned in the study of Singh and Pandey (2004), two of their study areas (Nagalapuram Hills and Darjeeling), which are similar to the physiography of our study area, have higher proportions of Papilionidae than other study areas (exceeding 10%). Thus, according to the reference proportion values of Palilionidae in the Central Himalayan subregions (like Nagalapuram Hills and Darjeeling of India), the estimated number of butterfly species in our study area would be 130 according to the model developed by Singh and Pandey (2004), which is closer to our present result of 138 species. On the other hand, our study might have a lower species richness estimate due to our shorter sampling period (one year) as compared to that of Nagalapuram Hills, which sampled butterflies over 1.5 years, though the exact sampling period of Darjeeling district was not mentioned. Finally, our study was conducted along the periphery of the wetland region only, which may help explain the different results found in this study, compared to those of previous studies conducted in forest habitats, due to differences in availability of host plants preferred by the species of particular butterfly families.

In our study, the Nymphalidae had the highest diversity followed by the Lycaenidae family. Previous studies have also reported Nymphalidae to have the highest species richness (Kunte, 1997; Prajapati et al., 2000; Shrestha et al., 2018; Tamang, Joshi, et al., 2019). Moreover, similar findings have also been reported in other wetlands in India, such as at Oussudu Lake (Murugesan et al., 2013) and the Kole Wetlands (Sarath et al., 2017). The rich biodiversity of butterfly fauna in Rupa Wetland is likely due to the moist climate due to high rainfall, abundant flowering plants which provides favorable habitat for the butterflies, and the moderate habitat disturbances creating microhabitats and ecological niches for harboring different species, all of which enhance diversity. Kumar Acharya and Vijayan, (2011) and Chettri et al. (2018) made similar observations during their study in Eastern Himalaya.

Interestingly, there was large variation in diversity among families. Some butterfly families observed in Rupa Wetland had quite high diversity (e.g., Nymphalidae), while others had lower diversity (i.e., Riodinidae and Hesperiidae), which may be due to a number of reasons. One possible explanation is that most nymphalids are polyphagous in nature, which makes it easier for them to utilize a variety of habitats (Janz, 2005). A second possible explanation may be that many species in this family are strong active fliers, which likely helps them cover large areas when searching for resources (Eswaran & Pramod, 2005; Padhye et al., 2006). Additionally, the families with relatively low diversity in Rupa Wetland may be limited by a lack of appropriate host plants; a study in South Germany found that the butterfly families with lower species richness were limited by a lack of host plants for the growing caterpillars (Steffan‐Dewenter & Tscharntke, 1997). The low sampling of species belonging to families Lycaenidae, Riodinidae, and Hesperidae might be due to the obscure habits of different species of butterflies under those families. Additionally, different butterfly families have different characteristics, which make some easier to spot and identify than others. For example, Papilionidae are active fliers, eye‐catching, and colorful, while Lycaenidae are small‐sized species that are difficult to identify in both flight and rest, as well as being unable to fly for long stretches and thus often landing on vegetation to rest, where they are less noticeable (Haribal, 1992). Furthermore, the timing of observations might be another reason for the fewer sightings of species under those families, since our study period was concentrated only in the morning between 8:00 to 12:00.

When examining all butterfly species pooled together, all three factors examined (plant category, flower color, and corolla type) were found to significantly influence butterfly visitation at nectar plants. Butterflies visited the flowers of herbaceous plant species significantly more often than the flowers of woody species. Similar to our results, a previous study in Japan found that nectar utilization by adult butterflies was substantially higher at herbaceous plants than at woody species, even though the study was conducted in and near a woodland (Kitahara et al., 2008). Additionally, a study by Santhosh and Basavarajappa (2016) found that weeds contributed the most nectar to butterfly species, followed by shrubs, herbs, trees, and climbers. Nimbalkar et al. (2011) also reported that visits of butterflies were more frequent to the flowers of herbs and shrubs than to the flowers of trees. This apparently common preference that butterfly species exhibit toward herbaceous plants may be due to the abundance of such host plants. For example, a study conducted by Sengupta and Ghorai (2013) in the hill forests of West Bengal, India found that Pierid and Hesperid butterfly families were mostly dependent on epiphytic flora due to the large availability of Ochideaceae plants within their study area. In our study area, the presence of both native and exotic ornamentals greatly enhanced the area’s floral diversity, which appears to benefit the native butterfly species. Because herbaceous species appear to offer attractive floral resources to butterflies, maintenance of herbaceous plants (particularly native species) in probable habitats may be one method to increase the richness and diversity of butterfly species.

In addition to plant category, flower color was also found to influence butterfly visitation. In our study, butterflies visited yellow, white, and purple flowers significantly more often than pink flowers. Previous studies examining floral color preferences in butterflies have reported a wide range of results. For example, one study in India found that butterflies visited red, yellow, blue, and purple flowers more often than white and pink flowers (Tiple et al., 2005). In contrast, a different study in India found that butterflies preferred yellow, white, pink, and blue flowers (Santhosh & Basavarajappa, 2016). The overall preference for yellow, white, and purple flowers in our study appears to be driven by Hesperidae, Lycaenidae, and Pieridae, as these families were rarely (0–1 individuals per transect) seen visiting pink or blue flowers (Figure 5). In contrast, Nymphalidae butterflies visited blue flowers more often than the other colors, and Papilionidae visited pink flowers most often, although the differences were not significant. Similar to our results, a previous study found that a Nymphalid butterfly species showed a color preference for both blue and yellow flowers (Ômura & Honda, 2005), and a different study reported that a Papilionidae species preferred red and purple flowers (Kandori & Yamaki, 2012). The diverse findings reported in previous studies is unsurprising given that there is high variation in floral rewards, both within and across plant species (Yan et al., 2016). Moreover, butterflies are known to be quick learners, and will readily choose high‐rewarding colors over innate color preferences (Blackiston et al., 2011; Kandori & Yamaki, 2012). Thus, further research is necessary to determine the innate and acquired color preferences of Nepalese butterflies.

The third trait examined, corolla type, revealed that butterflies visited tubular flowers significantly more often than nontubular flowers. Our results are similar to those of Tiple et al. (2005), who also found that butterflies in India visited tubular flowers more often than nontubular flowers. Moreover, the findings of Nimbalkar et al. (2011) also showed that most butterflies preferred tubular flowers over nontubular ones. Raju et al. (2004) reported butterflies feeding on both tubular and nontubular flowers, but exhibited a preference for tubular flowers. This generally universal preference that butterflies have for tubular flowers is unsurprising, given the suitable morphological fit between butterfly proboscises and tubular corolla tubes (Sultana et al., 2017).

Not only did we find that butterflies preferred tubular flowers, but we also observed a significant correlation between the proboscis length of butterflies and the corolla tube length of visited flowers. This finding indicates that butterflies with short proboscises prefer flowers with short corolla tube lengths and vice versa, although butterflies with long proboscises are still able to feed on flowers with shorter corolla tubes, as was observed in our data, resulting in a correlation coefficient of 0.466. At the same time, this finding supports the use of proboscis length as a morphological indicator of resource utilization in butterflies. Similar findings were recorded in the study by Corbet, (2000) which showed that the maximum corolla depth of potential nectar plants limits the species feeding on them to those with sufficiently long proboscises; short‐tongued butterfly species are therefore unable to feed on deep flowers. Moreover, Sultana et al. (2017) found that the proboscis had significant role in the coevolution between butterflies and their nectaring plants. They reported that flowers are only fed upon when they remain within the range of the proboscis length. Szigeti et al. (2020) investigated the relation between flower visits and the proboscis length of Clouded Apollo butterflies and found that the longer the proboscis, the more likely such butterflies were to forage on plants with the deepest corollas. Our study shows that Lycaenidae and Pieridae butterflies prefer flowers with relatively shallow corollas, Nymphalidae and Hesperidae with moderately deep corollas, and Papilionidae butterflies with the deepest corollas. A previous study by Tiple et al., (2009) in central India also showed that Papilionids foraged on flowers with long corolla tubes. Similarly, Ranta and Lundberg, (1980) reported that species with the longest proboscises were able to utilize the highest range of corolla tube depths. Thus, a long proboscis permits feeding on a greater variety of flowering plant species.

When analyzing butterfly visitation by family, Hesperidae were found to prefer yellow flowers over purple, and tubular flowers over nontubular, but for the remaining four families examined (Lycaenidae, Nymphalidae, Papilionidae, and Pieridae), none of the tested factors (plant category, flower color, and corolla type) were shown to significantly influence butterfly abundance at flowers.

Studying the relationship between butterflies and host plants in Nepal has important implications for conserving not only butterfly species, but also the host plants that they depend on, as well as the plants that depend on these butterflies for pollination. Such information is necessary to formulate effective conservation programs. Since there is positive correlation between the diversity of vegetation conditions and insect species diversity (Gardner et al., 1995), including butterfly diversity (Thomas, 1995), protection as well as cultivation of host plant species can help to enhance the conservation of butterflies in their respective ecosystems (Mukherjee et al., 2019). Similarly, diverse host plants, including cultivated species such as *Lantana camera*, in our study area provide rich sources of nectar for butterflies (Ramesh et al., 2010). Protection and maintenance of butterfly species diversity requires not only conservation of their primary habitat, but also the surrounding seminatural environments (Kitahara, 2004), which often primarily consist of herbs and shrubs (Shrestha et al., 2020). Thus, both forests and seminatural habitats are essential for the conservation of butterflies, and local stakeholders should be encouraged to protect such resources (Munyuli, 2013). Restoration of ecosystems helps to enable the rapid recovery of insect communities, which have generally decreased over time (Nyafwono et al., 2014), so the conservation of Rupa Wetland can provide a significant contribution to enhance butterfly species diversity.

## 5 CONCLUSIONS

Within the study area of Rupa Lake, 138 species belonging to 6 families were recorded. The most abundant family was Nymphalidae followed by Lycaenidae, Pieridae, and others. The Shannon diversity index was also highest for the family Nymphalidae. Among the species recorded, 61 of them were rare and an additional 45 species fell under the category “very rare.” This study also examined the different factors affecting the choice of nectar plants by Himalayan butterflies. Our findings show that plant category, flower color, corolla type, and corolla tube length all influenced butterfly foraging. Specifically, butterflies significantly preferred the flowers of herbaceous plants over woody plants, tubular flowers over nontubular flowers, and yellow, while and purple flowers over pink flowers. There was also a significant correlation between the proboscis length of butterflies and the corolla tube length of the visited flowers. Our study suggests that Rupa Lake is a resource enriched habitat for different butterfly species. This study not only confirms the importance of providing nectar resources for butterflies, but also reveals which types of resources are most appropriate for butterfly fauna. Cultivating native plant species preferred by butterflies will provide a more suitable habitat for these important pollinators. Finally, the high butterfly diversity found in the shrubland surrounding Rupa Lake reveals that the conservation this area, and similar areas, is necessary, and we recommend that such areas be declared as butterfly parks or butterfly zones to promote public awareness and conservation efforts.

## CONFLICT OF INTEREST

The authors declare no conflict of interest.

## AUTHOR CONTRIBUTION

**Bandana Subedi:** Conceptualization (equal); Data curation (equal); Formal analysis (equal); Investigation (equal); Methodology (equal); Writing‐original draft (equal); Writing‐review & editing (equal). **Alyssa B. Stewart:** Conceptualization (equal); Formal analysis (equal); Methodology (equal); Validation (equal); Writing‐review & editing (equal). **Bijaya Neupane:** Conceptualization (equal); Formal analysis (equal); Methodology (equal); Supervision (lead); Writing‐review & editing (equal). **Sudha Ghimire:** Data curation (equal); Investigation (equal). **Hari Adhikari:** Conceptualization (equal); Formal analysis (equal); Methodology (equal); Visualization (equal); Writing‐review & editing (equal).

### Open Research Badges

This article has been awarded Open Data, Open Materials, Preregistered Research Designs Badges. All materials and data are publicly accessible via the Open Science Framework at https://datadryad.org/stash/share/sl1Gxc2KGVXJVqFWj40UmuBRAUAnWYo0JEffP0meEw4; https://doi.org/10.5061/dryad.qnk98sfdg.

## Supporting information

Appendix S1‐S3

## Data Availability

Data are available on Dryad.URL: https://datadryad.org/stash/share/sl1Gxc2KGVXJVqFWj40UmuBRAUAnWYo0JEffP0meEw4; https://doi.org/10.5061/dryad.qnk98sfdg.
