# Supplementary material for: Butterfly species diversity and their floral preferences in the Rupa Wetland of Nepal
Source: Ecol Evol. 2021 Feb 3;11(5):2086–99. doi: 10.1002/ece3.7177 (PMC7920788; doi:10.1002/ece3.7177)
Supplement: Supplementary file 1 — Appendix S1‐S3 [file ECE3-11-2086-s001.docx]

# **Appendix**

Annex 1: Diversity of butterflies in Rupa Wetland, Nepal. Based on the number of sightings, butterfly species were categorized as very rare (< 2 sightings), rare (2–15 sightings), not rare (15–50 sightings), and common (50–100 sightings). Only a single individual of Common batwing (*Troides helena*) was recorded in the study area, even though this species was categorized under Least Concern according to the IUCN Red List Data, suggesting the need for more additional research in understudied areas such as Nepal.

| **Scientific Name** | **Local Name** | **Individuals** | **Family** | **Butterfly Status** |
| --- | --- | --- | --- | --- |
| *Lethe confuse* | Banded tree brown | 10 | Nymphalidae | Rare |
| *Ypthima baldus* | Common five ring | 45 | Nymphalidae | Not rare |
| *Ypthima nikaea* | Moore's five ring | 5 | Nymphalidae | Rare |
| *Ypthima huebneri* | Common four ring | 30 | Nymphalidae | Not rare |
| *Ypthima newara* | Newari three ring | 6 | Nymphalidae | Rare |
| *Ypthilma confuse* | Confusing three ring | 2 | Nymphalidae | Very rare |
| *Junonia almana* | peacock pansy | 12 | Nymphalidae | Rare |
| *Junonia lemonias* | Lemon pansy | 35 | Nymphalidae | Not rare |
| *Junonia orithyra* | Blue pansy | 2 | Nymphalidae | Very rare |
| *Junonia atlites* | Grey pansy | 80 | Nymphalidae | Common |
| *Junonia iphita* | Chocolate pansy | 27 | Nymphalidae | Not rare |
| *Tirmala septentrionis* | Dark blue tiger | 1 | Nymphalidae | Very rare |
| *Parantica tytia* | Chestnut tiger | 1 | Nymphalidae | Very rare |
| *Danaus genutia* | Common tiger | 5 | Nymphalidae | Rare |
| *Danaus chrysippus* | Plain tiger | 7 | Nymphalidae | Rare |
| *Parantica aglea* | Glassy tiger | 15 | Nymphalidae | Rare |
| *Euploea mulciber* | Striped blue crow | 3 | Nymphalidae | Rare |
| *Euploea core* | Common Indian Crow | 6 | Nymphalidae | Rare |
| *Hestina nama* | Circe | 2 | Nymphalidae | Very rare |
| *Elymnias hypermnestra* | Common palmfly | 1 | Nymphalidae | Very rare |
| *Elymnias malelas* | Spotted palmfly | 2 | Nymphalidae | Very rare |
| *Tanaecia lepidea* | Grey count | 29 | Nymphalidae | Not rare |
| *Tanaecia julii* | Common earl | 43 | Nymphalidae | Not rare |
| *Orsotrioena medus* | Jungle brown | 33 | Nymphalidae | Not rare |
| *Mycalesis francisca* | Lilacine bush brown | 7 | Nymphalidae | Rare |
| *Mycalesis malsara* | White line bush brown | 10 | Nymphalidae | Rare |
| *Mycalesis mineus* | Dark brand bushbrown | 5 | Nymphalidae | Rare |
| *Mycalesis perseus* | Common bush brown | 18 | Nymphalidae | Not rare |
| *Melanitis leda* | Common evening brown | 20 | Nymphalidae | Not rare |
| *Melanitis phedima* | Dark evening brown | 16 | Nymphalidae | Not rare |
| *Lethe insana* | Common forester | 1 | Nymphalidae | Very rare |
| *Nemetis mekara* | Straight red forester | 1 | Nymphalidae | Very rare |
| *Argyreus hyperbius* | Indian fritillary | 6 | Nymphalidae | Rare |
| *Symbrenthia niphanda* | Blue tailed jester | 1 | Nymphalidae | Very rare |
| *Symbrenthia hypselis* | Spotted jester | 1 | Nymphalidae | Very rare |
| *Symbrenthia lilaea* | Common jester | 10 | Nymphalidae | Rare |
| *Pantoporia hordonia* | Common Lascar | 8 | Nymphalidae | Rare |
| *Euthalia aconthea* | Common baron | 11 | Nymphalidae | Rare |
| *Neptis hylas* | Common sailor | 29 | Nymphalidae | Not rare |
| *Neptis cartica* | Plain sailor | 7 | Nymphalidae | Rare |
| *Neptis sankara* | Broad banded sailor | 2 | Nymphalidae | Very rare |
| *Neptis zaida bhutanica* | Pale green sailor | 1 | Nymphalidae | Very rare |
| *Kallima inachus* | Orange oakleaf | 4 | Nymphalidae | Rare |
| *Doleschallia bisaltide* | Autumn leaf | 1 | Nymphalidae | Very rare |
| *Cyrestis thyodamas* | Common map | 5 | Nymphalidae | Rare |
| *Chersonesia risa* | Common maplet | 9 | Nymphalidae | Rare |
| *Cupha erymanthis* | Rustic | 1 | Nymphalidae | Very rare |
| *Phalanta phalantha* | Common leopard | 15 | Nymphalidae | Rare |
| *Polyura athamas* | Common nawab | 2 | Nymphalidae | Very rare |
| *Aglais cashmirensis* | Indian tortoiseshell | 2 | Nymphalidae | Very rare |
| *Limenitis danava* | Common commodore | 3 | Nymphalidae | Rare |
| *Ariadne merione* | Common castor | 15 | Nymphalidae | Rare |
| *Hypolimnas bolina* | Great eggfly | 4 | Nymphalidae | Rare |
| *Vanessa indica* | Indian red admiral | 6 | Nymphalidae | Rare |
| *Vanessa cardui* | Painted lady | 2 | Nymphalidae | Very rare |
| *Athyma nefte* | Colour sergeant | 1 | Nymphalidae | Very rare |
| *Athyma selenophora* | Staff sergeant | 1 | Nymphalidae | Very rare |
| *Athyma ranga* | Blackvein sergeant | 2 | Nymphalidae | Very rare |
| *Athyma perius* | Common sergeant | 12 | Nymphalidae | Rare |
| *Abrota ganga* | Sergeant major | 1 | Nymphalidae | Very rare |
| *Cethosia biblis* | Leopard lacewing | 3 | Nymphalidae | Rare |
| *Vagrans egista* | Vagrant | 5 | Nymphalidae | Rare |
| *Papilio polytes* | Common Mormon | 22 | Papilionidae | Not rare |
| *Papilio helenus* | Red Helen | 19 | Papilionidae | Not rare |
| *Atrophaneura aidoneus* | Common batwing | 1 | Papilionidae | Very rare |
| *Papilio protenor* | Spangle | 8 | Papilionidae | Rare |
| *Papilio bianor* | Common Peacock | 3 | Papilionidae | Rare |
| *Papilio paris* | Paris Peacock | 8 | Papilionidae | Rare |
| *Graphium sarpedon* | Common Bluebottle | 3 | Papilionidae | Rare |
| *Papilio memnon* | Great Mormon | 12 | Papilionidae | Rare |
| *Troides helena* | Common birdwing | 1 | Papilionidae | Very rare |
| *Pachliopta aristolochiae* | Common Rose | 1 | Papilionidae | Very rare |
| *Graphium chirnoides* | Veined Jay | 2 | Papilionidae | Very rare |
| *Graphium agamemnon* | Tailed Jay | 9 | Papilionidae | Rare |
| *Papilio machaon* | Common Yellow Swallowtail | 1 | Papilionidae | Very rare |
| *Pseudocoladenia dan* | Fulvous Pied Flat | 19 | Hesperiidae | Not rare |
| *Tagiades menaka* | Spotted snow flat | 8 | Hesperiidae | Rare |
| *Tagiades litigiosa* | Water Snow Flat | 2 | Hesperiidae | Very rare |
| *Notocrypta curvifascia* | Restricted Demon | 3 | Hesperiidae | Rare |
| *Telicota bambusae* | Dark palm Dart | 22 | Hesperiidae | Not rare |
| *Parnara guttata* | Straight swift | 69 | Hesperiidae | Common |
| *Sarangesa dasahara* | Common Small Flat | 23 | Hesperiidae | Not rare |
| *Iambrix salsala* | Chestnut bob | 15 | Hesperiidae | Rare |
| *Ochus subvittatus* | Tiger hopper | 1 | Hesperiidae | Very rare |
| *Spialia galba* | Indian skipper | 1 | Hesperiidae | Very rare |
| *Catopsilia pomona* | Common Emigrant | 12 | Pieridae | Rare |
| *Catopsilia pyranthe* | Mottled Emigrant | 16 | Pieridae | Not rare |
| *Pieris cannida* | Indian Cabbage White | 4 | Pieridae | Rare |
| *Pieris brassicae* | Large Cabbage White | 2 | Pieridae | Very rare |
| *Hebomoia glaucippe* | Great Orange Tip | 5 | Pieridae | Rare |
| *Appias lyncida* | Chocolate albatross | 4 | Pieridae | Rare |
| *Eurema andersonii* | One spot grass yellow | 5 | Pieridae | Rare |
| *Eurema blanda* | Three-spot-grass Yellow | 29 | Pieridae | Not rare |
| *Eurema hecabe* | Common Grass Yellow | 38 | Pieridae | Not rare |
| *Cepora nadina* | Lesser Gull | 14 | Pieridae | Rare |
| *Cepora nerissa* | Common Gull | 12 | Pieridae | Rare |
| *Delias hyparete* | Painted jezabel | 5 | Pieridae | Rare |
| *Delias acalis* | Red Breast jezabel | 1 | Pieridae | Very rare |
| *Delias eucharis* | Common jezabel | 1 | Pieridae | Very rare |
| *Delias descombesi* | Red-spot Jezabel | 22 | Pieridae | Not rare |
| *Delias pasithoe* | Red-Base Jezabel | 5 | Pieridae | Rare |
| *Pareronia avatar* | Pale wanderer | 2 | Pieridae | Very rare |
| *Gandaca harina* | Tree yellow | 1 | Pieridae | Very rare |
| *Ixias pyrene* | Yellow orange tip | 2 | Pieridae | Very rare |
| *Belonois aurota* | Pioneer | 1 | Pieridae | Very rare |
| *Dodona egeon* | Orange Punch | 5 | Riodinidae | Rare |
| *Abisara neophron* | Tailed Judy | 30 | Riodinidae | Not rare |
| *Dodona adonira* | Striped punch | 5 | Riodinidae | Rare |
| *Zemeros flegyas* | Punchinello | 92 | Riodinidae | Common |
| *Jamides celeno* | Common Cerulean | 34 | Lycaenidae | Not rare |
| *Jamides alecto* | Metallic Cerulean | 30 | Lycaenidae | Not rare |
| *Zizina otis* | Lesser Grass Blue | 22 | Lycaenidae | Not rare |
| *Zizeeria karsandra* | Dark Grass Blue | 12 | Lycaenidae | Rare |
| *Zizeeria maha* | Pale Grass Blue | 10 | Lycaenidae | Rare |
| *Arhopala paramuta* | Hooked Oakblue | 23 | Lycaenidae | Not rare |
| *Jamides bochus* | Dark Cerulean | 5 | Lycaenidae | Rare |
| *Arhopala amantes* | Large Oakblue | 2 | Lycaenidae | Very rare |
| *Arhopala centaurus* | Centaur Oakblue | 1 | Lycaenidae | Very rare |
| *Rapala nissa* | Common Flash | 20 | Lycaenidae | Not rare |
| *Castalius rosimon* | Common Pierrot | 7 | Lycaenidae | Rare |
| *Tarucus ananda* | Dark Pierrot | 8 | Lycaenidae | Rare |
| *Curetis bulis* | Bright sunbeam | 5 | Lycaenidae | Rare |
| *Spindiasis syama* | Club silverline | 1 | Lycaenidae | Very rare |
| *Spindasis lohita* | Long-brand silverline | 2 | Lycaenidae | Very rare |
| *Everes lacturnus* | Indian Cupid | 9 | Lycaenidae | Rare |
| *Prosotas nora* | Common lineblue | 15 | Lycaenidae | Rare |
| *Prosotas dubiosa* | Tailless lineblue | 20 | Lycaenidae | Not rare |
| *Heliophorus epicles* | Purple Sapphire | 14 | Lycaenidae | Rare |
| *Acytolepsis puspa* | Common hedge blue | 23 | Lycaenidae | Not rare |
| *Lestranicus transpecta* | White banded hedge blue | 6 | Lycaenidae | Rare |
| *Lampides boeticus* | Pea blue | 3 | Lycaenidae | Rare |
| *Catochrysops strabo* | Forget-me-not blue | 2 | Lycaenidae | Very rare |
| *Loxura atymnus* | Yamfly | 1 | Lycaenidae | Very rare |
| *Zeltus amasa* | Fluffy tit | 1 | Lycaenidae | Very rare |
| *Rapala pheritima* | Copper flash | 24 | Lycaenidae | Not rare |
| *Rapala manea* | Slate flash | 12 | Lycaenidae | Rare |
| *Chliaria othona* | Orchid tit | 2 | Lycaenidae | Very rare |
| *Anthene emolus* | Ciliate Blue | 5 | Lycaenidae | Rare |

Annex 2: Observations of butterfly species at nectar plants, the proboscis length of butterflies, and relevant traits of nectar plants (plant category, flower colour, corolla type, and corolla length). T= Tubular and NT= Non-tubular

| **S.N** | **Butterfly**  **species** | **Scientific Name** | **Proboscis**  **length**  **(mm)** | **Plant species** | **Plant**  **category** | **Flower**  **colour** | **Corolla type** | **Corolla**  **length**  **(mm)** |
| --- | --- | --- | --- | --- | --- | --- | --- | --- |
| 1 | Red Admiral | *Vanessa indica* | 18.2 | *Lantana camara* | woody | yellow | T | 12 |
| 2 | Common tiger | *Danaus genutia* | 10 | *Eupatorium odoratum* | woody | purple | T | 9 |
| 3 | Glassy tiger | *Parantica aglea* | 13 | *Parthenium hysterophorus* | herb | white | T | 3 |
| 4 | Plain Tiger | *Danaus chrysippus* | 13 | *Lantana camara* | woody | yellow | T | 12 |
| 5 | Red Admiral | *Vanessa indica* | 18.2 | *Bidens pilosa* | herb | white | T | 5 |
| 6 | Indian Fritilary | *Argyreus hyperbius* | 12 | *Zinnia elegans* | herb | pink | T | 2 |
| 7 | Common tiger | *Danaus genutia* | 13 | *Sida rhambifolia* | herb | yellow | NT | 3 |
| 8 | Common jester | *Symbrenthia lilaea* | 13 | *Lantana camara* | woody | yellow | T | 12 |
| 9 | Chocolate Pansy | *Junonia iphita* | 13 | *Ageratina adenophora* | herb | purple | T | 3 |
| 10 | Peacock pansy | *Junonia almana* | 11.4 | *Lantana camara* | woody | yellow | T | 12 |
| 11 | Grey pansy | *Junonia atlites* | 13 | *Ageratum conyzoides* | herb | white | T | 3 |
| 12 | Grey pansy | *Junonia atlites* | 13 | *Ageratum houstonianum* | herb | blue | T | 4 |
| 13 | Lemon pansy | *Junonia lemonias* | 12 | *Bidens pilosa* | herb | white | T | 5 |
| 14 | Common tiger | *Danaus genutia* | 8 | *Lantana camara* | woody | yellow | T | 12 |
| 15 | Striped blue crow | *Euploea mulciber* | 9.3 | *Eupatorium odoratum* | herb | purple | T | 9 |
| 16 | Common indian crow | *Euploea core* | 12 | *Ageratum houstonianum* | herb | blue | T | 4 |
| 17 | Common indian crow | *Euploea core* | 12 | *Tagetes erecta* | herb | yellow | T | 17 |
| 18 | Common sailor | *Neptis hylas* | 7 | *Ageratina adenophora* | herb | purple | T | 3 |
| 19 | Common tiger | *Danaus genutia* | 10 | *Ageratum houstonianum* | herb | blue | T | 4 |
| 20 | Glassy tiger | *Parantica aglea* | 13 | *Bidens pilosa* | herb | white | T | 5 |
| 21 | Glassy tiger | *Parantica aglea* | 13 | *Ageratum houstonianum* | herb | blue | T | 4 |
| 22 | Plain Tiger | *Danaus chrysippus* | 13 | *Bidens pilosa* | herb | white | T | 5 |
| 23 | Common five ring | *Ypthima baldus* | 5.1 | *Borreria latifolia* | herb | purple | T | 4 |
| 24 | Peacock pansy | *Junonia almana* | 11.4 | *Eupatorium odoratum* | woody | purple | T | 9 |
| 25 | Plain Tiger | *Danaus chrysippus* | 11 | *Ageratum houstonianum* | herb | purple | T | 4 |
| 26 | Striped blue crow | *Euploea mulciber* | 9.3 | *Ageratum houstonianum* | herb | purple | T | 4 |
| 27 | Common indian crow | *Euploea core* | 12 | *Bidens pilosa* | herb | white | T | 5 |
| 28 | Common indian crow | *Euploea core* | 12 | *Zinnia elegans* | herb | pink | T | 6 |
| 29 | Common five ring | *Ypthima baldus* | 5.1 | *Bidens pilosa* | herb | white | T | 5 |
| 30 | Lemon pansy | *Junonia lemonias* | 12 | *Cuphea hyssopifolia* | woody | purple | T | 8 |
| 31 | Lemon pansy | *Junonia lemonias* | 12 | *Lantana camara* | woody | yellow | T | 12 |
| 32 | Lemon pansy | *Junonia lemonias* | 12 | *Eupatorium odoratum* | herb | purple | T | 9 |
| 33 | Club silverline | *Spindiasis syama* | 8 | *Eupatorium odoratum* | woody | purple | T | 9 |
| 34 | Common cerulian | *Jamides celeno* | 6.1 | *Vitex negundo* | woody | purple | T | 5 |
| 35 | Copper flash | *Rapala pheritima* | 9 | *Eupatorium odoratum* | woody | purple | T | 9 |
| 36 | Copper flash | *Rapala pheritima* | 9 | *Bidens pilosa* | herb | white | T | 5 |
| 37 | Pea blue | *Lampides boeticus* | 7 | *Tridax procumbens* | herb | white | T | 8 |
| 38 | Club silverline | *Spindiasis syama* | 8 | *Lantana camara* | woody | yellow | T | 12 |
| 39 | Common pierrot | *Castalius rosimon* | 8 | *Sida rhambifolia* | herb | yellow | NT | 3 |
| 40 | Indian cupid | *Everes lacturnus* | 4 | *Bidens pilosa* | herb | white | T | 5 |
| 41 | Indian cupid | *Everes lacturnus* | 4 | *Desmodium confertum* | herb | purple | T | 4 |
| 42 | Pea blue | *Lampides boeticus* | 7 | *Duranta erecta* | woody | purple | T | 7 |
| 43 | Common pierrot | *Castalius rosimon* | 8 | *Bidens pilosa* | herb | white | T | 5 |
| 44 | Great Mormon | *Papilio memnon* | 34 | *Eupatorium odoratum* | woody | purple | T | 9 |
| 45 | Common mime | *Chilasa clytia* | 14 | *Lantana camara* | woody | yellow | T | 12 |
| 46 | Common mormon | *Papilio polytes* | 25 | *Ipomoea quamoclit* | herb | pink | T | 26 |
| 47 | Common mormon | *Papilio polytes* | 25 | *Mussaenda roxburghii* | woody | yellow | T | 28 |
| 48 | Spangle | *Papilio protenor* | 32 | *Salvia coccinea* | herb | red | T | 29 |
| 49 | Common mormon | *Papilio polytes* | 25 | *Lantana camara* | woody | yellow | T | 12 |
| 50 | Common mormon | *Papilio polytes* | 25 | *Bougainvillea glabra* | woody | pink | T | 20 |
| 51 | Mottled Emigrant | *Catopsilia pyranthe* | 16 | *Bidens pilosa* | herb | white | T | 5 |
| 52 | Mottled Emigrant | *Catopsilia pyranthe* | 16 | *Zinnia elegans* | herb | pink | T | 2 |
| 53 | Common grass yellow | *Eurema hecabe* | 15 | *Parthenium hysterophorus* | herb | white | T | 3 |
| 54 | Red spot jezeble | *Delias descombesi* | 16 | *Sambucus adnata* | herb | white | NT | 4 |
| 55 | Chocolate Albatross | *Appias lyncida* | 14 | *Bidens pilosa* | herb | white | T | 5 |
| 56 | Mottled Emigrant | *Catopsilia pyranthe* | 16 | *Eupatorium odoratum* | woody | purple | T | 9 |
| 57 | Chocolate Albatross | *Appias lyncida* | 14 | *Sambucus wightiana* | herb | white | NT | 4 |
| 58 | Common grass yellow | *Eurema hecabe* | 15 | *Borreria latifolia* | herb | purple | T | 4 |
| 59 | Common grass yellow | *Eurema hecabe* | 15 | *Eupatorium odoratum* | woody | purple | T | 9 |
| 60 | Mottled Emigrant | *Catopsilia pyranthe* | 16 | *Bidens pilosa* | herb | white | T | 5 |
| 61 | Common grass yellow | *Eurema hecabe* | 15 | *Sida rhambifolia* | herb | yellow | NT | 3 |
| 62 | Red spot jezeble | *Delias descombesi* | 16 | *Eupatorium odoratum* | herb | purple | T | 9 |
| 63 | Red spot jezeble | *Delias descombesi* | 16 | *Castanopsis indica* | woody | white | NT | 4 |
| 64 | Common grass yellow | *Eurema hecabe* | 15 | *Tagetes erecta* | herb | yellow | T | 17 |
| 65 | Mottled Emigrant | *Catopsilia pyranthe* | 16 | *Zinnia elegans* | herb | yellow | T | 6 |
| 66 | Chocolate Albatross | *Appias lyncida* | 14 | *Eupatorium odoratum* | herb | purple | T | 9 |
| 67 | Chocolate Albatross | *Appias lyncida* | 14 | *Lantana camara* | woody | yellow | T | 12 |
| 68 | Straight Swift | *Parnara guttata* | 17 | *Sida rhambifolia* | herb | yellow | NT | 3 |
| 69 | Straight Swift | *Parnara guttata* | 17 | *Borreria latifolia* | herb | purple | T | 4 |
| 70 | Common small flat | *Sarangesa dasahara* | 12 | *Parthenium hysterophorus* | herb | white | T | 3 |
| 71 | Himalayan spotted flat | *Celaenorrbinus munda* | 20 | *Urena lobata* | herb | pink | NT | 12 |
| 72 | Straight Swift | *Parnara guttata* | 17 | *Bidens pilosa* | herb | white | T | 5 |
| 73 | Straight Swift | *Parnara guttata* | 17 | *Ageratum conyzoides* | herb | white | T | 3 |
| 74 | Common small flat | *Sarangesa dasahara* | 12 | *Borreria latifolia* | herb | purple | T | 4 |
| 75 | Straight Swift | *Parnara guttata* | 17 | *Coreopsis lanceolata* | herb | yellow | T | 4 |
| 76 | Straight Swift | *Parnara guttata* | 17 | *Cosmic sulphureus* | herb | yellow | T | 3 |
| 77 | Straight Swift | *Parnara guttata* | 17 | *Mimosa pudica* | herb | purple | NT | 2 |
| 78 | Straight Swift | *Parnara guttata* | 17 | *Cosmic sulphureus* | herb | yellow | T | 3 |
| 79 | Straight Swift | *Parnara guttata* | 17 | *Acmella uliginosa* | herb | yellow | T | 4 |
| 80 | Straight Swift | *Parnara guttata* | 17 | *Duranta erecta* | woody | purple | T | 7 |

Annex 3. Descriptive statistics (by family) for butterfly proboscis length (in mm) and corolla tube length (in mm) of flowers visited by butterflies

|  | **Family** | **Number** | **Mean** | **Standard deviation** | **Median** | **Inter quartile range** | **Range** |
| --- | --- | --- | --- | --- | --- | --- | --- |
| **Proboscis length (mm)** | Hesperidae | 13 | 16.46 | 2.15 | 17 | 0 | 12–20 |
|  | Nymphalidae | 32 | 11.63 | 2.80 | 12 | 2.25 | 5.1–18.2 |
|  | Pieridae | 16 | 15.18 | 0.83 | 15 | 1.25 | 14–16 |
|  | Lycaenidae | 11 | 7.10 | 1.75 | 8 | 1.45 | 4–9 |
|  | Papilionidae | 7 | 25.71 | 6.42 | 25 | 3.5 | 14–34 |
| **Corolla tube length (mm)** | Hesperidae | 13 | 4.38 | 2.60 | 4 | 1 | 2–12 |
|  | Nymphalidae | 32 | 6.69 | 3.84 | 5 | 5 | 2–17 |
|  | Pieridae | 16 | 6.63 | 3.96 | 5 | 5 | 2–17 |
|  | Lycaenidae | 11 | 6.55 | 2.70 | 5 | 3.5 | 3–12 |
|  | Papilioni  dae | 7 | 19.43 | 8.44 | 20 | 15 | 9–29 |

Annex 4. Photographs of butterfly species observed in Rupa Lake. a. ***Vanessa indica*** feeding on *Lantana Camara* b. ***Argynnis hyperbius*** on *Zinnia elegans* c. ***Danaus genutia*** on *Sida rhambifolia* d. ***Junonia iphita*** on *Ageratum adenophora* e. ***Junonia almana*** on *Lantana camara* f. ***Junonia lemonias*** on *Bidens pilosa* g. ***Euploea mulciber*** on *Ageratum conyzoides* h. ***Euploea core*** on *Ageratum conyzoides* I. ***Euploea core*** on *Zinnia elegans*  j. ***Parantica aglea*** in *Ageratum conyzoides* k. ***Spindasis lohita*** on *Lantana camara* l. ***Danaus chrysippus*** on *Bidens pilosa* m. ***Junonia almana*** on *Lantana camara* n. ***Danaus chrysippus*** on *Ageratum conyzoides* o. ***Junonia lemonias*** on *Lantana camara* p. ***Delias*** ***pasithoe*** on *Eupatorium odoratum* q. ***Danaus genutia*** on *Ageratum conyzoides* r. ***Junonia lemonias*** on *Cuphea hyssopifolia*  s. ***Vanessa cardui*** on *Ageratum adenophora* t. ***Eurema*** ***andersoni*** on *Urena lobata* u. ***Delias descombesi*** on *Lantana camara* v. ***Everes lacturnu***s on *Desmodium confertum* w. ***Catopsilia pomona*** on *Zinnia elegans* x. ***Tagaides litigiosa*** on *Lantana camara* y. ***Papilio memon*** on *Eupatorium odoratum* z. ***Papilio protenor*** on *Salvia coccinea*

| 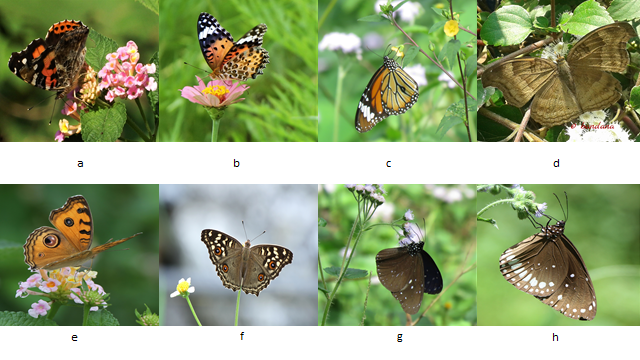 |
| --- |
| 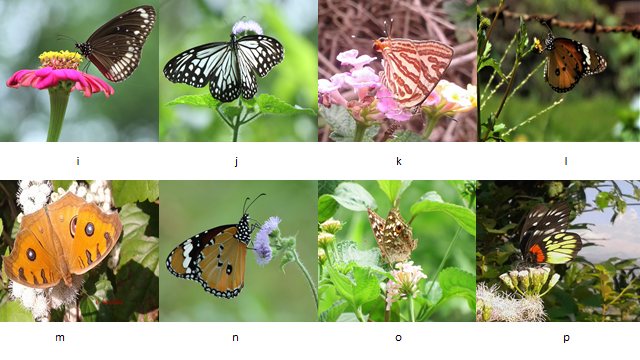 |
| 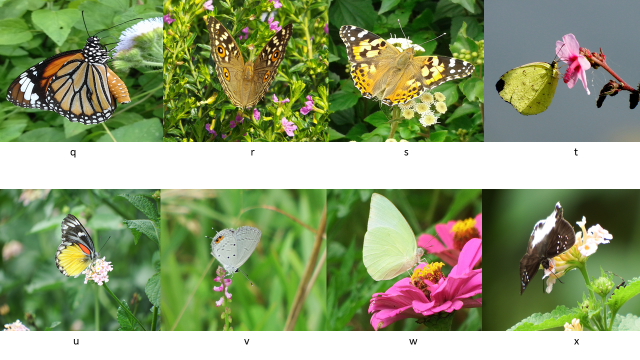 |
| 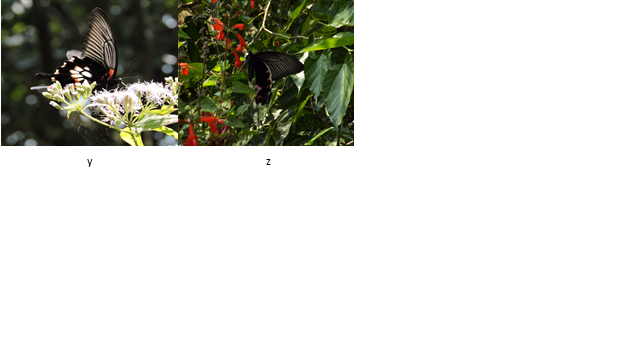 |
